# Effects of Anemoside B4 on Diarrhea Incidence, Serum Indices, and Fecal Microbial of Suckling Calves

**DOI:** 10.3389/fvets.2022.851865

**Published:** 2022-04-28

**Authors:** Meng Lu, Fengming Hu, Yanliang Bi, Tao Ma, Qiyu Diao, Linshu Jiang, Yan Tu

**Affiliations:** ^1^Key Laboratory of Feed Biotechnology of Ministry of Agriculture and Rural Affairs, Institute of Feed Research of Chinese Academy of Agricultural Sciences, Beijing, China; ^2^Beijing Key Laboratory for Dairy Cow Nutrition, Beijing University of Agriculture, Beijing, China

**Keywords:** Anemoside B4, suckling calves, diarrhea, serum indices, fecal microbial

## Abstract

The study was conducted to evaluate the effects of Anemoside B4 on diarrhea incidence, serum indices, and fecal microbial of suckling calves. Sixty newborn Chinese Holstein calves with similar body weight (43.7 ± 3.9 kg) were randomly divided into four groups with 15 calves each, fed the diet which was supplied 0 (CON), 15 (A1), 30 (A2), and 45 (A3) mg/day of Anemoside B4, respectively. The trial period is 56 days. The blood and fecal samples were collected at 28 and 56 days of age. Results show that during the whole trial period, the diarrhea incidence in Group A1, A2, and A3 was significantly lower than that in Group CON (*p* < 0.05). Compared with the Group CON, Anemoside B4 supplementation significantly decreased the contents of serum D-lactic acid and diamine oxidase at 28-day-old (*p* < 0.05). At 56-day-old, the content of serum D-lactic acid in Group A3 tended to be higher (0.05 < *p* < 0.01), and the content of serum diamine oxidase in Group A3 increased significantly, in comparison with Group CON (*p* < 0.05). Group A3 increased the level of Chao1 and Simpson indices at 28-day-old (0.05 < *p* < 0.01), and Chao1, Observed_species, Shannon, and Simpson indices at 56-day-old (*p* < 0.05), in comparison to Group CON. Compared with Group CON, 45 mg / day Anemoside B4 supplementation significantly increased the contents of *Bacteroidota* (at the phylum level), *Prevotella* (at the genus level) at 28-day-old (*p* < 0.05), and the content of *Sutterella* (at the genus level) at 56-day-old (*p* < 0.05), promoted the processes of energy metabolism, glycan biosynthesis and metabolism, metabolism of cofactors and vitamins (*p* < 0.05). A positive correlation was observed between *Prevotella* and metabolism of cofactors and vitamins, energy metabolism, and glycan biosynthesis and metabolism. A positive correlation was observed between *Sutterella* and energy metabolism. In conclusion, Anemoside B4 could effectively alleviate calf diarrhea, protect the integrity of intestinal mucosa, and change the structure of intestinal microbiota, indicating the potential value of Anemoside B4 in regulating intestinal microbiota and the prevention of intestinal diseases.

## Introduction

Calve breeding is related to the quality and production level of the herd, which in turn affects the production efficiency of the entire cattle industry (1). Newborn calves are susceptible to disease, and diarrhea is the main cause of death in suckling calves, which has extremely high morbidity and mortality (2). According to the statistics of the U.S. National Animal Health Monitoring System study, published in 2018, 39% of calf mortality was caused by diarrhea in the first 3 weeks after birth (3), which is mainly caused by intestinal injury and incomplete establishment of intestinal flora in calves (2, 4, 5). Among them, *Escherichia coli* (*E.coli*), *Bovine viral diarrhea virus, B. coronavirus*, and *B. rotavirus* are the main pathogens that cause diarrhea in calves (6). The growth of pathogens in calves reduces the number of beneficial bacteria, which greatly increases the risk of disease (7). In addition, damage to the intestine will cause the increase of intestinal permeability, the invasion of the pathogen, the destruction of the intestinal immune barrier, the inflammatory reaction of the intestine tract, the decrease of calf immunity, and the diarrhea of calves (8).

Some plants contain a variety of active ingredients such as saponins, polysaccharides, and essential oils, which play important roles in inhibiting the growth of pathogens, improving immunity, and promoting intestinal health without toxic and side effects (9). Plant extracts are secondary metabolites of plants (10), which have the functions of antimicrobial (11), and anti-inflammatory (12), and have been proposed as substitutes for chemical feed additives with the banning of some ionophore feed additives in many countries (13).

Pulsatilla Radix, derived from the dry roots of Pulsatilla Chinensis Regel in Ranunculaceae, has a history of thousands of years growing in China and plays an important role in antibacterial, anti-inflammatory, and immune enhancement (14, 15). Saponins are a class of complex glycoside compounds, which are the basis for many plants to exert their pharmacological effects. Anemoside is the main component of Pulsatillae Radix and the basis for their anti-inflammatory and anti-tumor biological activities. The Anemoside in Pulsatillae Radix mainly includes Anemoside B4, Anemoside A3, and 23-hydroxy betulinic acid. Among them, Anemoside B4, a natural triterpenoid glycoside isolated, is the monomer component with the highest content in Pulsatillae Radix, which has antibacterial and anti-inflammatory effects (16). *In vitro* antibacterial test reveals that Anemoside B4 can inhibit the growth of pathogenic bacteria such as *E. coli* (17). Meanwhile, Anemoside B4 can inhibit the expression of pro-inflammatory factors such as IL-1β, IL-6, and IL-8, and enhance the anti-inflammatory ability of the body (18). In addition, a study proved that Anemoside B4 at 30 ml/q. d. I. M. in brachiocephalic muscle for 4–6 days can provide a largely side-effect-free cure for cows with clinical mastitis (19).

A few studies have demonstrated the antibacterial activity and therapeutic potential of triterpenoid saponin Anemoside B4, but its application in calves has not been reported. Thus, this study was conducted to study the effect of Anemoside B4 on diarrhea incidence, serum intestinal permeability indicators, and fecal microbes of suckling calves by adding Anemoside B4 into milk replacer, to provides the theoretical basis for the development of feed additives.

## Materials and Methods

### Experimental Design, Animal Management, and Diet

The feeding trial was carried out at the Nankou Base of the Institute of Feed Research, Chinese Academy of Agricultural Science (IFRCAAS) (Beijing, China) from March to May 2021. The experimental procedures were approved by the Animal Ethics Committee of IFRCAAS (AEC-IFR-CAAS, Beijing, China).

Sixty healthy newborn Holstein bull calves (body weight = 43.7 ± 3.9 kg; the serum total protein concentration was >55g/L) from Shou Nong Group Co., Ltd. (Beijing, China) were selected for the experiment. The calves were removed from their dams shortly after birth and fed colostrum in a transfer room. Each calf consumed a total of 6 L colostrum, with 4 L fed within 2 h of life and the remaining 2 L fed 8 h after the first feeding. Then all calves were moved to a naturally ventilated hutch and kept in individual pens (4.5 × 1.5 m). The experiment lasted 56 days with 14 days for the preliminary feeding period and 42 days for a formal trial period. All calves were fed with commercial MR (Beijing Precision Animal Nutrition Research Center, Beijing, China) for the transition from 7 to 14 days of age, and then randomly assigned to one of the four groups (15 calves per group) at 14 days of age based on body weight and age.

The milk replacer is fed at 1.25% (dry matter basis) of body weight (at 07:00, 13:00, and 19:00) and adjusted fortnightly. The MR was mixed with boiling water and cooled to 50–60°C to form an emulsion at a ratio of 1:7 (weight/volume) and then fed to calves when its temperature was lowered to 38°C. Anemoside B4 was added to the milk replacer during morning feeding. The calves of the four groups were fed the diet with 0 (CON), 15 (A1), 30 (A2), and 45 (A3) mg/day per head Anemoside B4, respectively. The starter feed and fresh water were provided *ad libitum* throughout the study.

The milk replacer powder, configured in accordance with the national invention patent CN 02128844.5, was provided by Beijing Precision Animal Nutrition Center (Beijing, China), and the nutrient levels are shown in Supplementary Table 1. The starter feed was provided by Sanyuan Hefeng Animal Husbandry Co., Ltd. (Beijing, China), and the ingredients and compositions are shown in Supplementary Table 2. The Anemoside B4 (content 67.41%) was purchased from Nanjing Chunqiu Biological Engineering Company., Ltd. (Jiangsu, China).

### Diarrhea Incidence

Observe the health of the calves every day and score the fecal by the four-point system. The fecal scoring standard is shown in Table 1 (20). Whenever an animal presented a fecal score ≥3 for 2 consecutive days, they were considered diarrheic, and diarrhea incidence was calculated as follow:

**Table 1:** Fecal score standard.

**Items**	**Outward**	**Dry matter (%)**	**Score**
Normal	Bars or granules	>30	1
Mild	Soft manure, can be formed	25–30	2
Moderate	Thick, shapeless, no separation of manure, and water	20–25	3
Serious	Liquid, not shaped, separation of manure, and water	<20	4

Diarrhea incidence (%) = total number of diarrhea calves × 100 / (number of calves in the group × trial days).

### Sample Collection

At 28 and 56 days of age, 2 h after morning feeding, six healthy calves in each group whose body weight was close to the average body weight of the group, were selected. Blood samples were collected from the jugular vein and centrifuged at 2,000 *g* for 10 min. The collected serum was placed in a 1.5 ml centrifuge tube and frozen at −20°C for further analysis. The concentrations of D-lactic acid (D-LA) and diamine oxidase (DAO) in serum samples were analyzed with commercial kits from Nanjing Jiancheng Bioengineering Institute (Jiangsu, China) according to the enzyme-labeler (ST-360). In addition, fecal samples were collected by rectal fecal collection method, divided into 1.5 ml cryopreservation tubes, and quickly placed in liquid nitrogen for preservation, and then brought back to the laboratory for cryopreservation at −80°C for DNA extraction and identification of fecal microbial.

### DNA Extraction and 16S rRNA Pyrosequencing

DNA was extracted using the kit, and the V3-V4 region of 16S rDNA was amplified with specific primers with barcode, primer sequence was 338F: ACTCCTACGGGAGGCAGCAG; 806 r: GGACTACHVGGGTWTCTAAT. PCR products were detected by 1% agarose gel electrophoresis and purified with Agencourt AMPure XP nucleic acid purification kit. The purified amplified products were mixed in equal quantities and connected to the “Y” shaped connector. The self-connecting segments of the connector were removed by magnetic bead screening. The library template was enriched by PCR amplification to generate single-stranded DNA fragments, and the MiSeq library was constructed and sequenced by MiSeq.

### Data Processing

The original data contains certain low-quality data, which easily affects the subsequent data analysis. To ensure the reliability and biological validity of the subsequent analysis, the original data needs to be preprocessed. Firstly, Trimmomatic (V 0.36) and Pear (V 0.9.6) were used to quality control the original data and obtain the Fasta sequence. Uchime method was used to compare and remove the chimera of the Fasta sequence according to the known database, while denovo method was used to remove the chimera of the Fasta sequence for the unknown database. At the same time, the unqualified short sequences were removed to obtain the high-quality sequence clean_tags. Qiime software (Version 1.8.0) was used to conduct statistical analysis of bioinformatics on OTU at a 97% similar level. Richness estimates and diversity indices including Chao 1, Observed_species, Goods_coverage, PD_whole_tree, Shannon, and Simpson were calculated using the QIIME V1.8. To obtain the species classification information corresponding to each OTU, BLAST (2.6.0+) was used for comparative analysis of OTU representative sequences, and the specific information of the community was annotated at various levels. linear discriminant analysis (LDA) effect size analysis was used to find species with significant differences in abundance between each group (21). First, the ANOVA test was used to detect species with significant differences in abundance between different groups. Then, the Wilcoxon rank-sum test was used to analyze the differences between different groups. Finally, LDA was used to reduce the data and assess the impact of species with significant differences, and the algebraic linear discriminant analysis score was set to ≥3.0. A PCoA based on the weighted UniFrac distances was conducted to compare all samples, and a distance-based matrices analysis was performed to evaluate differences among samples.

### Microbial Function Prediction

Use phylogenetic investigation of communities by reconstruction of unobserved states version 2.0.0 to predict the function of the fecal microbial community (PICRUSt v 2.0.0; http://galaxy.morganlangille.com) (22). The information of KO, Pathway, EC was obtained according to the Kyoto Encyclopedia of Genes and Genomes analysis module and calculated the abundance of each functional category.

### Statistical Analysis

The χ2 procedure was used to compare the incidence of diarrhea between treatment groups. The fecal score was conducted using GraphPad Prism Version 9 (GraphPad Software Inc. CA, USA). The data of serum indices, alpha diversity indices, the relative abundance of microbial flora, and metabolism level were analyzed by the independent sample *t*-test, and all statistical analyses were performed by using SAS software (version 9.4, SAS Institute Inc., Cary, NC, USA). The ‘ggcor' package GitHub of R was used to analyze the relationship between fecal microbial and metabolism. Treatment differences with *p* < 0.05 were considered statistically significant and 0.05 < *p* < 0.10 was designated as a tendency. Some or all data, models, or code generated or used during the study are available from the corresponding author by request.

## Results

### Diarrhea Incidence

As shown in Table 2, compared with Group CON, Anemoside B4 significantly decreased the diarrhea incidence of calves throughout the experiment (*p* < 0.05), and that in Group A3 decreased significantly in comparison to Group A1(*p* < 0.05). From 7 to 42 days of age, the incidence of diarrhea in Group A1, A2, and A3 was significantly lower compared to Group CON (*p* < 0.05). Moreover, the diarrhea incidence of calves in Group A1 and A3 was significantly lower at 29 to 42 days of age in comparison with that in Group CON (*p* < 0.05). There was no significant difference among the four groups at 43–56 days (*p* > 0.05).

**Table 2:** Effects of Anemoside B4 on diarrhea incidence of suckling calves (%).

	**Groups**				**χ2**	
**Items**	**CON**	**A1**	**A2**	**A3**		***p-value***
7 to 56 d	27.38^a^	20.62^b^	18.77^bc^	15.69^c^	29.18	<0.01
7 to 14 d	60.58^a^	43.27^b^	40.38^b^	35.58^b^	14.95	0.002
15 to 28 d	31.32^a^	20.88^b^	15.38^b^	14.84^b^	19.51	<0.01
29 to 42 d	28.02^a^	14.84^b^	17.58^b^	12.64^b^	16.96	0.001
43 to 56 d	8.24	7.14	7.69	4.40	2.49	0.477

*CON, the dosage of B4 is 0 mg / d; A1, the dosage of B4 is 15 mg / day; A2, the dosage of B4 is 30 mg / day; A3, the dosage of B4 is 45 mg / day; SEM, Standard error of the mean*.

abc *Denotes a diversity trait with significant difference (p < 0.05)*.

### Serum Profiles

Table 3 shows that Anemoside B4 increased the content of serum D-lactic acid (D-LA) and diamine oxidase (DAO) at 28 days of age (*p* < 0.05), and D-LA and DAO levels gradually increased with the addition of Anemoside B4. At 56 days of age, D-LA in Group A3 tended to be higher than that in Group CON (0.05 < *p* < 0.10), and the Group A3 had a higher level of DAO than Group CON (*p* < 0.05).

**Table 3:** Effects of Anemoside B4 on serum indices of suckling calves.

	**Groups**				**SEM**	
**Items**	**C**	**A1**	**A2**	**A3**		***p*** **-Valve**
**28 days of age**						
D-LA, nmol / L	11.87^a^	11.08^b^	10.77^b^	9.92^c^	0.176	<0.01
DAO, ng / mL	1.21^a^	1.06^b^	0.96^b^	0.84^c^	0.034	<0.01
**56 days of age**						
D-LA, nmol / L	12.81	12.46	12.47	12.23	0.110	0.08
DAO, ng / mL	1.41^a^	1.39^ab^	1.34^ab^	1.27^b^	0.024	0.04

*CON, the dosage of B4 is 0 mg / d; A1, the dosage of B4 is 15 mg / day; A2, the dosage of B4 is 30 mg / day; A3, the dosage of B4 is 45 mg / day; SEM, Standard error of the mean; D-LA, D-lactic acid; DAO, diamine oxidase*.

abc *Denotes a diversity trait with significant difference (p < 0.05)*.

### Sequencing Depth and Index of Microbial Community

**Figure 1:**
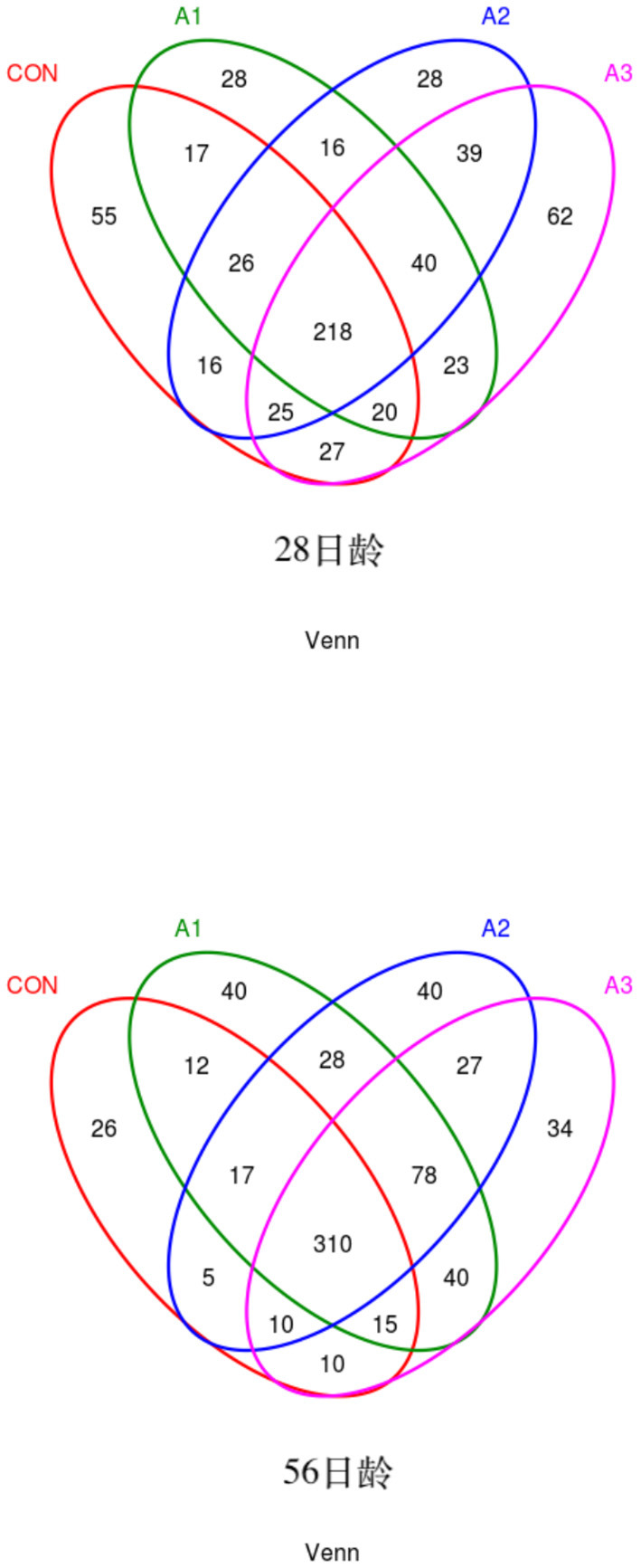
Venn diagram illustrating overlap of microbial OTUs at 3% dissimilarity level among treatments. Venn diagram of bacteria at 28 days of age (left) or 56 days of age (right). CON, the dosage of B4 is 0 mg / day; A1, the dosage of B4 is 15 mg / day; A2, the dosage of B4 is 30 mg / day; A3, the dosage of B4 is 45 mg / day.

**Figure 2:**
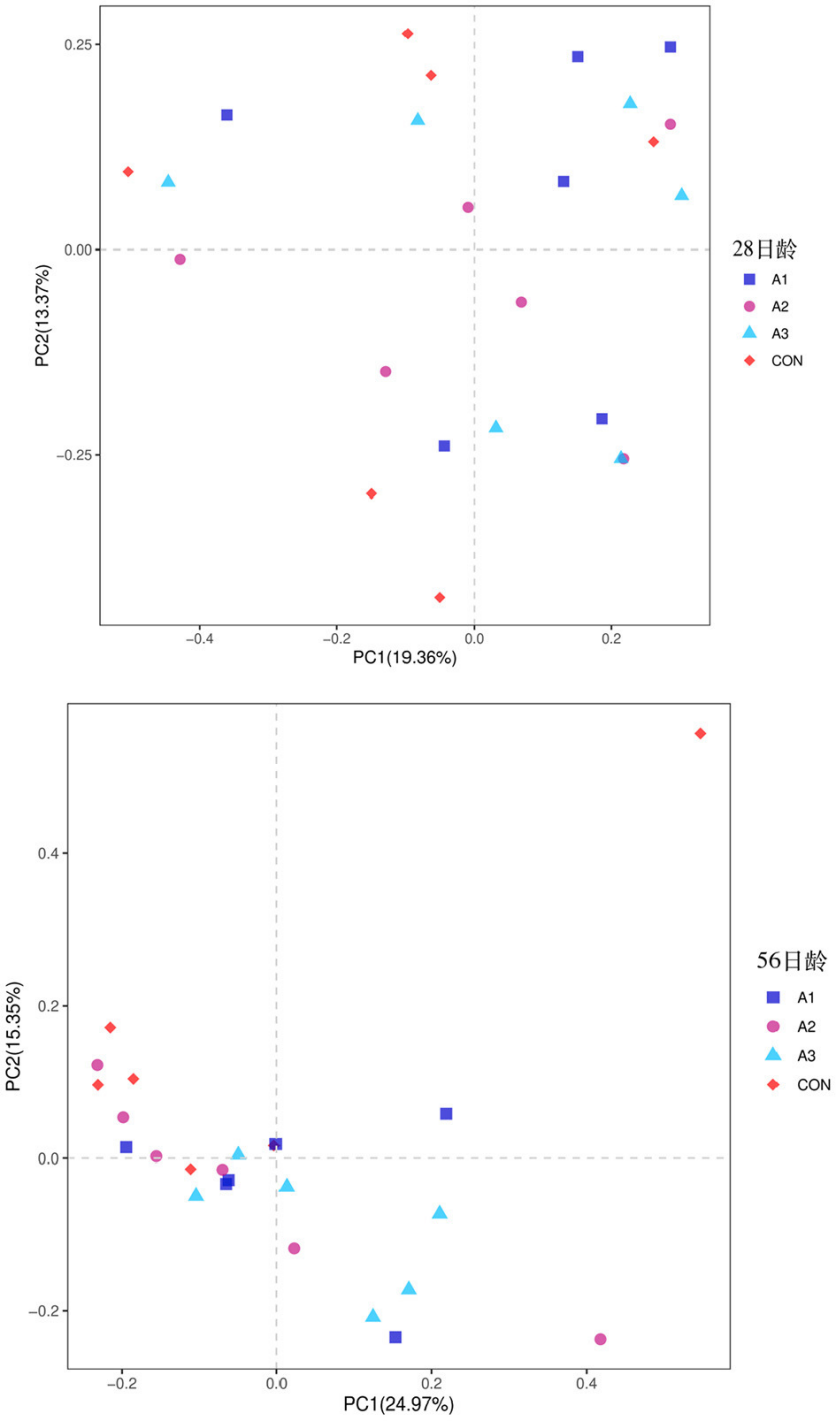
Principal Coordinate Analysis (PCoA) of fecal bacterial community structures of suckling calves in the four groups at 28 days of age (left) and 56 days of age (right). CON, the dosage of B4 is 0 mg / day; A1, the dosage of B4 is 15 mg / day; A2, the dosage of B4 is 30 mg / day; A3, the dosage of B4 is 45 mg / day.

**Figure 3:**
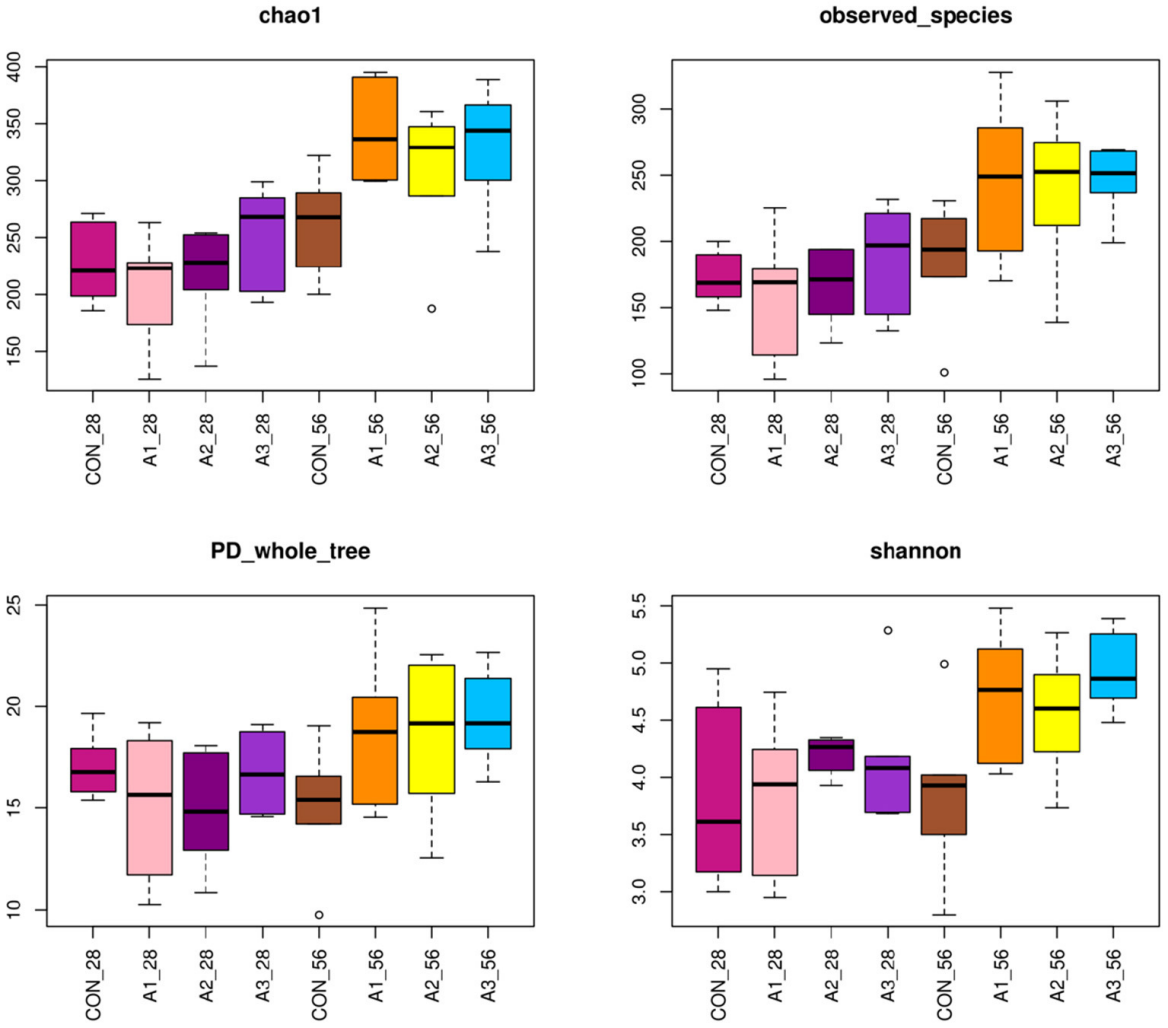
Richness estimates and diversity indices for bacteria with four groups. The top and bottom boundaries of each box represent the 75th and 25th quartile values, respectively. The horizontal lines inside each box represent the median values. CON, the dosage of B4 is 0 mg / day; A1, the dosage of B4 is 15 mg / day; A2, the dosage of B4 is 30 mg / day; A3, the dosage of B4 is 45 mg / day.

The fecal microbes of calves in the four groups were sequenced based on 16S rDNA technology. After the original data were further removed from the chimera and short sequences, a total of 1,383,096 and 1,007,665 high-quality sequences were obtained at 28 and 56 days of age, accounting for 90.00 % and 93.43% of the original data, respectively. As shown in Figure 1, a total of 1,654 OUTs were identified, of which a total of 55, 28, 28, and 62 OUTs were unique in Group CON, A1, A2, and A3, respectively, at 28 days of age, and a total of 1,984 OUTs were identified, of which 405, 540, 515, and 524 OUTs were identified in Group CON, A1, A2, and A3 at 56 days of age, respectively. Based on the weighted UniFrac and bray-Curtis distance matrix, principal coordinate analysis (PCoA) was further used to analyze the structure of the microbial community in calf fecal as shown in Figure 2. Through the distance of the samples, the differences between individuals or groups could be observed. The greater the distance between the samples, the greater the difference in the composition of the microbial community between the samples. PCoA plots of bacterial 16S rRNA showed that the data points of the calf stool sample at 28 days of age were relatively scattered, and the different treatments were not well-clustered, indicating that there was a serious microbial imbalance in the intestinal tract of calves with diarrhea. At 56 days of age, the sample points were well-clustered, and the four groups could be largely separated as shown in Figure 2. In addition, the Chao1 indices of fecal microbial in Group A3 tended to be higher in comparison with that in Group A1 at 28 days of age (0.05 < *p* < 0.10), which in Group CON decreased by 11.44% in Figure 3. The PD_ whole_Tree and Shannon indices showed no marked differences among the four groups (*p* > 0.05), while Simpson indices in Group A2 showed a higher trend than that in Group CON (0.05 < *p* < 0.10). At 56 days of age, the alpha diversity indices in Group A3, and the Shannon and Simpson indices in Group A2 increased significantly compared with Group CON (*p* < 0.05).

### Bacterial Composition of Fecal Microbial Flora Across Different Treatments

There were 14 phylum and 203 genera were identified in fecal microbes. As shown in Tables 4, 5, the main bacteria at the phylum level among four groups were *Firmicutes, Bacteroidota*, followed by *Fusobacteriota, Actinobacteriota*, and *Proteobacteria*. *Megasphaera* and *Prevotella* were dominant bacteria at the genus level in Tables 6, 7. At the age of 28 days, the abundance of *Bacteroidota* at phylum level in Group A3 (23.17%) was significantly higher than that in Group CON (1.78%) and Group A1 (4.35%). Compared with Group CON, the *Euryarchaeota* abundance in Group A1 (0.05%) and *Desulfobacterota* abundance in Group A3 (0.01%) tended to increase in Table 4 (0.05 < *p* < 0.10). At the genus level, *Prevotella* abundance in Group CON (0.91%) and A1 (1.91%) (*p* < 0.05) decreased significantly compared with Group A3 (12.21%), and there is a trend of significant difference of *Sutterella* abundance among Group A3 (1.72%), Group A1 (0.19%), and A2 (0.25%) (0.05 < *p* < 0.10). The *Mitsuokella* abundance of the calf fecal microflora in Group A2 (4.49%) tended to be higher in comparison to Group CON (0.74%) in Table 5 (0.05 < *p* < 0.10). As shown in Table 6, at 56 days of age, no significant differences were observed in fecal microbial flora community and relative abundance among four groups (*p* > 0.05), among which the abundance of *Bacteroidota* in Group CON tended to be higher than that in Group A1 (0.05 < *p* < 0.10), while *Firmicutes* abundance tended to be lower (0.05 < *p* < 0.10). Besides, the abundance of *Proteobacteria* and *Desulfobacterota* in Group A3 was higher than those in Group CON (0.05 < *p* < 0.10). At the genus level, there were no significant differences among the four groups except for *Prevotella, Faecalibacterium, Acidaminococcus, Dialister*, and *Sutterella* as shown in Table 7. Compared with Group CON, *Prevotella* in Group A1 and A3 tended to decrease (0.05 < *p* < 0.10), *Faecalibacterium* and *Sutterella* in Group A3 and *Acidaminococcus* in Group A1 increased significantly (*p* < 0.05), and *Dialister* in Group A2 showed a trend to increase (0.05 < *p* < 0.10).

**Table 4:** Effects of Anemoside B4 on the comparison of dominant phylum of suckling calves at 28 days of age (%).

**Phylum**	**Diets**				**SEM**	***p*** **-value**
	**CON**	**A1**	**A2**	**A3**		
*Firmicutes*	78.93	73.63	71.90	62.55	0.042	0.60
*Proteobacteria*	5.15	16.47	7.80	5.87	0.028	0.47
*Actinobacteriota*	5.99	5.42	4.33	2.25	0.008	0.44
*Verrucomicrobia*	0.31	0.00	0.00	0.01	0.001	0.42
*Bacteroidetes*	1.78^b^	4.35^b^	12.57^ab^	23.17^a^	0.032	0.02
*Fusobacteriota*	7.75	0.02	3.32	5.97	0.023	0.70
*Desulfobacterota*	0.01	0.03	0.02	0.11	0.000	0.09
*Euryarchaeota*	0.05	0.00	0.02	0.00	0.000	0.06
*Campilobacterota*	0.02	0.01	0.01	0.01	0.000	0.86
*Synergistota*	0.01	0.01	0.01	0.03	0.000	0.54
*Patescibacteria*	0.00	0.02	0.02	0.01	0.000	0.72
*Spirochaetota*	0.00	0.00	0.00	0.01	0.000	0.42
*Cyanobacteria*	0.00	0.04	0.00	0.00	0.000	0.43
*Acidobacteriota*	0.00	0.00	0.00	0.00	0.000	0.41

*CON, the dosage of B4 is 0 mg / d; A1, the dosage of B4 is 15 mg / d; A2, the dosage of B4 is 30 mg / d; A3, the dosage of B4 is 45 mg / d; SEM, Standard error of the mean*.

ab *Denotes a diversity trait with significant difference (p < 0.05)*.

**Table 5:** Effects of Anemoside B4 on the comparison of dominant phylum of suckling calves at 56 days of age (%).

**Phylum**	**Diets**				**SEM**	***p*** **-value**
	**CON**	**A1**	**A2**	**A3**		
*Bacteroidota*	53.08	35.32	45.44	37.88	0.034	0.07
*Firmicutes*	43.99	59.20	49.97	52.08	0.030	0.09
*Proteobacteria*	1.68	1.81	2.42	4.47	0.005	0.06
*Actinobacteriota*	1.18	3.26	2.02	5.15	0.008	0.37
*Desulfobacterota*	0.05	0.30	0.05	0.28	0.000	0.07
*Synergistota*	0.01	0.02	0.01	0.03	0.000	0.38
*Cyanobacteria*	0.00	0.04	0.03	0.02	0.000	0.37
*Campilobacterota*	0.00	0.00	0.00	0.00	0.000	0.41
*Verrucomicrobia*	0.00	0.00	0.00	0.01	0.000	0.39
*Euryarchaeota*	0.00	0.00	0.00	0.00	0.000	0.41
*Spirochaetota*	0.00	0.02	0.01	0.03	0.000	0.64
*Patescibacteria*	0.00	0.04	0.01	0.03	0.000	0.36
*Fusobacteriota*	0.00	0.00	0.03	0.03	0.000	0.60

*CON, the dosage of B4 is 0 mg / d; A1, the dosage of B4 is 15 mg / d; A2, the dosage of B4 is 30 mg / d; A3, the dosage of B4 is 45 mg / day; SEM, Standard error of the mean*.

**Table 6:** Effects of Anemoside B4 on the comparison of dominant genus of suckling calves at 28 days of age (%).

**Genus**	**Diets**				**SEM**	***p*** **-value**
	**CON**	**A1**	**A2**	**A3**		
Megasphaera	15.62	22.44	13.97	20.18	0.032	0.79
Prevotella	0.91^b^	1.91^b^	8.44^ab^	12.21^a^	0.016	0.03
Parasutterella	0.90	0.60	2.42	3.28	0.006	0.40
Dialister	3.42	2.69	6.12	2.88	0.011	0.67
Romboutsia	2.46	0.05	0.23	2.25	0.007	0.44
Faecalibacterium	10.66	5.27	5.52	7.51	0.026	0.89
Mitsuokella	0.74	3.92	4.49	2.11	0.007	0.08
Megamonas	1.13	7.46	9.14	6.40	0.022	0.62
Bacteroides	0.11	0.48	2.04	4.34	0.010	0.49
Fusobacterium	7.75	0.02	3.32	5.97	0.023	0.70
Clostridium_sensu_stricto_1	2.88	1.25	0.93	3.41	0.008	0.67
Olsenella	2.18	0.57	1.65	1.69	0.005	0.68
Lachnospiraceae_NK3A20_Group	3.08	2.41	0.77	2.06	0.008	0.77
Sutterella	0.53^ab^	0.19^b^	0.25^b^	1.72^a^	0.003	0.04
Alloprevotella	0.34	1.62	1.82	5.04	0.011	0.50

*CON, the dosage of B4 is 0 mg / d; A1, the dosage of B4 is 15 mg / d; A2, the dosage of B4 is 30 mg / d; A3, the dosage of B4 is 45 mg / d; SEM, Standard error of the mean*.

ab *Denotes a diversity trait with significant difference (p < 0.05)*.

**Table 7:** Effects of Anemoside B4 on the comparison of dominant genus of suckling calves at 56 days of age (%).

**Genus**	**Diets**				**SEM**	***p*** **-value**
	**CON**	**A1**	**A2**	**A3**		
Prevotella	50.65	30.97	36.4	30.68	0.037	0.06
Megasphaera	15.79	16.92	20.05	14.54	0.020	0.82
Faecalibacterium	2.06^b^	3.58^ab^	4.72^ab^	7.84^a^	0.010	0.04
Acidaminococcus	4.53^b^	13.39^a^	6.7^ab^	4.94^b^	0.014	0.02
Bifidobacterium	0.35	1.77	1.09	3.47	0.007	0.44
Dialister	1.64	1.88	4.58	3.04	0.005	0.06
Bacteroides	0.46	1.05	2.33	2.84	0.007	0.64
Subdoligranulum	0.58	3.15	2.32	2.63	0.006	0.43
Megamonas	0.79	1.59	0.09	2.11	0.006	0.64
Sutterella	0.28^b^	0.42^b^	0.46^b^	2.05^a^	0.003	0.02
Mitsuokella	2.3	2.27	3.56	1.8	0.004	0.52
uncultured	1.09	1.32	1.95	1.59	0.003	0.72
Alloprevotella	0.93	1.31	3.18	1.58	0.007	0.72
Escherichia-Shigella	0.58	0.45	0.79	1.47	0.003	0.57
Phascolarctobacterium	0.5	0.61	0.4	1.41	0.002	0.47

*CON, the dosage of B4 is 0 mg / d; A1, the dosage of B4 is 15 mg / d; A2, the dosage of B4 is 30 mg / d; A3, the dosage of B4 is 45 mg / day; SEM, Standard error of the mean*.

ab *Denotes a diversity trait with significant difference (p < 0.05)*.

**Figure 4:**
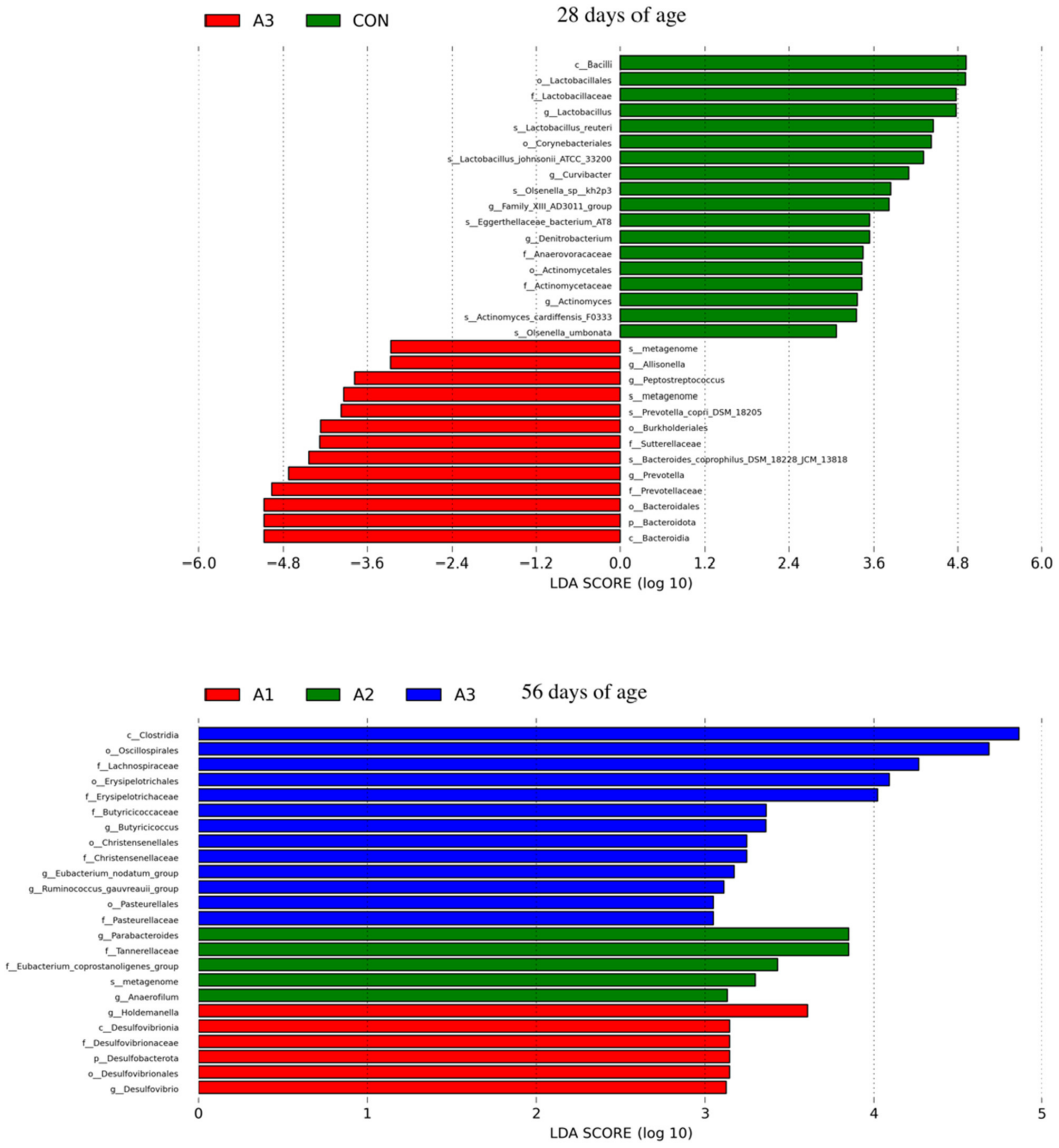
The potential biomarkers were defined by LEfSe at 28 and 56 days of age. The significantly different taxa are signified by different colors representing the four groups. Histogram of the LDA scores for differentially abundant features among groups. The threshold on the logarithmic LDA score for discriminative features was set to 3.0. CON, the dosage of B4 is 0 mg / day; A1, the dosage of B4 is 15 mg / day; A2, the dosage of B4 is 30 mg / day; A3, the dosage of B4 is 45 mg / day.

The potential biomarkers of different groups are presented by LEfSe as shown in Figure 4. The microbial with the LDA score > 3 is a specific microbial, which distinguishes one group from other groups. At 28 days of age, a total of 33 specific microbial genera (LDA score >3) were found in Group A3 and CON, in which the *c_Bacilli, o_Lactobacillales, f_Lactobacillaceae* were enriched in Group CON, and *c_Bacteroidia, p_Bacteroidota, o_Bacteroidales* were enriched in Group A3. At 56 days of age, a total of 24 specific microbial genera (LDA score >3) were found in Group A1, A2, and A3, among which the *c_Clostridia, o_Osicillospirales*, and *f_Lachnospiraceae* were enriched in Group A3. These genera had a significant impact on the sample Grouping.

### Predictive Analysis of Bacterial Community Function

Before 28 days of age, the diarrhea incidence of calves in each group was significantly different. Phylogenetic Investigation of Communities by Reconstruction of Unobserved States two was used to predict the function of marker genes in the four treatments groups at 28 days of age, indicating that it was mainly concentrated in metabolism, genetic information processing, environmental information processing, and cellular processes and organic systems. The relative abundance of metabolism and genetic information processing is relatively high. The genetic information processing was similar among the four groups (*p* > 0.05), while metabolism showed a great difference (*p* < 0.05). Among these, carbohydrate metabolism in Group A1 and A2 was significantly higher than that in Group A3 (*p* < 0.05), while no obvious differences were found between Group CON and other groups (*p* > 0.05). The lipid metabolism and xenobiotics biodegradation and metabolism in Group CON were significantly higher than those in Group A2 and A3 (*p* < 0.05), while the metabolism of cofactors and vitamins and energy metabolism showed the opposite. In addition, Group A3 had a higher abundance of glycan biosynthesis and metabolism than other groups (*p* < 0.05) in Table 8.

**Table 8:** Effects of Anemoside B4 on metabolism of suckling calves at 28 days of age (%).

**Genus**	**Diets**				**SEM**	***p*** **-value**
	**CON**	**A1**	**A2**	**A3**		
Carbohydrate metabolism	14.87^ab^	15.65^a^	15.33^a^	14.30^b^	0.182	<0.01
Lipid metabolism	5.85^a^	5.05^b^	4.31^b^	4.18^b^	0.229	0.02
Metabolism of cofactors and vitamins	12.30^b^	12.97^ab^	13.75^a^	14.17^a^	0.260	0.04
Energy metabolism	4.97^b^	5.07^ab^	5.23^a^	5.30^a^	0.049	0.02
Nucleotide metabolism	2.22	2.14	2.15	2.20	0.031	0.80
Amino acid metabolism	12.44	12.59	13.31	13.17	0.203	0.36
Metabolism of terpenoids and polyketides	10.26	9.36	9.78	10.13	0.187	0.34
Biosynthesis of other secondary metabolites	2.11	2.01	1.80	2.00	0.082	0.62
Xenobiotics biodegradation and metabolism	2.96^a^	2.70^ab^	1.55^b^	1.17^b^	0.272	0.02
Metabolism of other amino acids	7.48	7.61	7.33	7.34	0.098	0.73
Glycan biosynthesis and metabolism	3.48^b^	3.82^ab^	4.37^ab^	4.92^a^	0.239	0.03

*CON, the dosage of B4 is 0 mg / d; A1, the dosage of B4 is 15 mg / day; A2, the dosage of B4 is 30 mg / d; A3, the dosage of B4 is 45 mg / d; SEM, Standard error of the mean*.

abc *Denotes a diversity trait with significant difference (P < 0.05)*.

### Correlation Between Fecal Microbial and Metabolism

**Figure 5:**
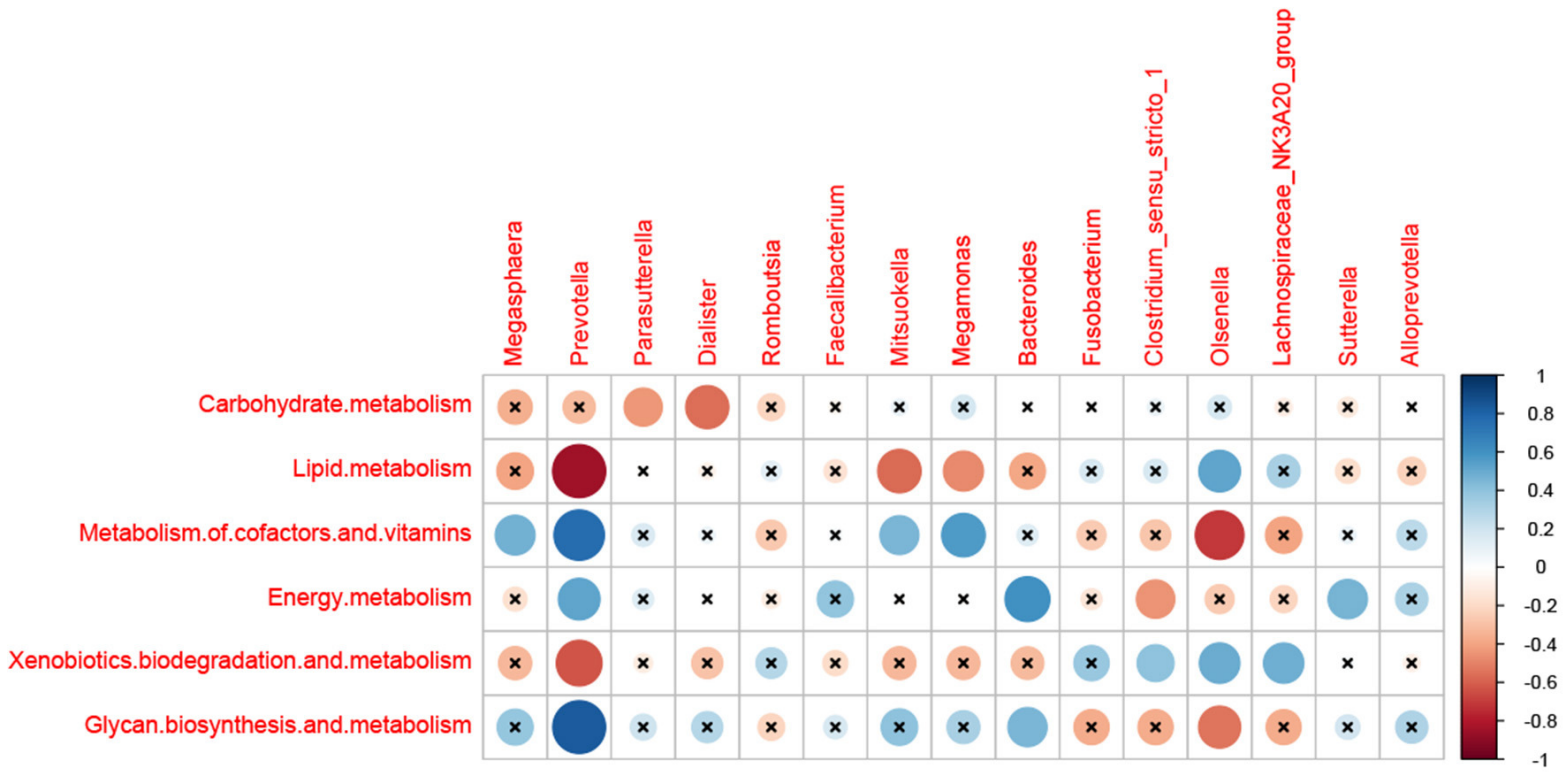
Correlation matrix between the metabolism and the dominant microbial at 28 days of age. The abscissa is the differential flora, and the ordinate is the differential metabolisms. Blue is positive and red is negative.

As shown in Figure 5, the relationships between the dominant microbial and metabolism were evaluated in this study. Carbohydrate metabolism was negatively correlated with *Parasutterella* and *Dialister* (*p* < 0.05). The lipid metabolism was negatively correlated with *Prevotella, Mitsuokella*, and *Megamonas* (*p* < 0.05), while positively correlated with *Olsenella* (*p* < 0.01). The Metabolism of cofactors and vitamins was positively correlated with *Megasphaera, Prevotella, Mitsuokella*, and *Megamonas* (*p* < 0.05), and negatively correlated with *Olsenella* (*p* < 0.01). Energy metabolism has a positive correlation with *Prevotella, Bacteroides*, and *Sutterella* (*p* < 0.05), and a negative correlation with *Clostridium_sensu_stricto_1* (*p* < 0.05). Xenobiotics biodegradation and metabolism was negatively correlated with *Prevotella* (*p* < 0.05), and positively correlated with *Clostridium_sensu_stricto_1, Olsenella*, and *Lachnospiraceae_NK3A20_group* (*p* < 0.05). Glycan biosynthesis and metabolism were positively correlated with *Prevotella* and *Bacteroides* (*p* < 0.05), and negatively correlated with *Olsenella* (*p* < 0.05), respectively.

## Discussion

*In vitro* antibacterial test showed that the extract of Pulsatillae Radix has a certain inhibitory effect on *Staphylococcus aureus, Shigella dysenteriae*, and *Salmonella typhi* (23). In this study, feeding 45 mg/day Anemoside B4 could reduce the diarrhea incidence of calves from 7 to 56-day-old, especially before 42 days of age, indicated that Anemoside B4 might have a direct inhibitory effect on the pathogenic bacteria of diarrhea, which is consistent with the results of the previous study (24). After 42 days of age, there was no significant difference in the incidence of diarrhea among the groups. This was mainly due to the gradual improvement of the calf's own immune system and the gradual stabilization of the intestinal flora, which can effectively resist the invasion of external pathogenic bacteria. The effect of Anemoside B4 on diarrhea gradually decreases with the age of calves increasing, which also indicates that the prevention and treatment effect of Anemoside B4 on the diarrhea of calves are mainly effective before 42 days of age.

The occurrence of diarrhea of suckling calves is mainly related to the immune status, and newborn calves with low immunity are prone to be affected by external pathogens (25). In a previous study, Anemoside B4 could significantly increase the content of IgG in the serum of suckling calves, indicating that Anemoside B4 could improve the immune ability of calves (26). In addition, diarrhea is associated with intestinal injury and disturbance of intestinal flora in calves (3, 4). The increase of intestinal permeability and the disorder of intestinal microbiota are the key factors leading to diarrhea caused by pathogens (27). DAO is a highly active cytoplasmic enzyme found in mammalian intestinal epithelial cells, and serum D-LA is a metabolite of gastrointestinal flora. Serum D-LA and DAO levels are negatively correlated with the integrity and maturity of the intestinal mucosa, so its level can indirectly reflect the changes in intestinal mucosal permeability and the degree of damage to the intestinal mechanical barrier, and it is also an important indicator for evaluating the integrity of the intestinal mucosa (28). When a disease occurs in the body, the intestinal permeability increases and a large amount of DAO and D-LA enter the blood circulation with the increase in intestinal permeability, resulting in an increase in serum DAO and D-LA levels. In this study, at 28 days of age, the serum D-LA and DAO levels of the calves without Anemoside B4 treatment are significantly higher than others, indicating that they are most likely to have intestinal mucosa diseases, such as injury and increased permeability. These also proved that Anemoside B4 has the effect of protecting the intestinal mucosa of suckling calves, and it is mainly achieved by reducing the permeability of the intestinal mucosa and improving the integrity of the intestinal mucosa (8). Therefore, Anemoside B4 can not only directly inhibit the reproduction of diarrhea-causing bacteria, but also can effectively reduce the permeability and damage of the intestinal mucosa of calves.

Anemoside B4 had notable anti-inflammation activity, and it could inhibit the expression of pro-inflammatory cytokines and enhance immunity (18). Newborn calves have low immunity, they need to obtain nutrients from the external environment to improve their own immunity for achieving the effect of active immunity (29). But it is also susceptible to the stimulation of various external environments to the disturbance of the calf intestinal flora, which leads to the occurrence of diarrhea. The complex microbiome plays a very important role in the barrier function of the intestinal mucosa and the growth and development of the body (30), and its composition is one of the important factors that determine whether a calf has diarrhea or not. The more diversified microorganisms are, the more beneficial it is to the health of the body. It is also considered to be a sign of the maturity of the intestinal microbial community (31), but the diversified microflora is not necessarily beneficial to the health of the body. The structure of the microbial community will have an adverse effect on the body's development (32). In this study, Anemoside B4 can improve the abundance and diversity of fecal microbial, mainly because low dose saponins can promote the penetration of cell membranes, make bacterial cells absorb more nutrients, promote the growth of bacterial (33), and reduce the number of protozoa (34, 35).

A healthy intestinal microbial can regulate host nutrition and intestinal development, digest nutrients that are difficult for the host to digest and absorb, produce metabolites required for host growth and development, enhance the host immune system by enhancing the interaction between antigens and immune cells, and prevent the colonization of foreign pathogens (27, 36). Therefore, microbial can also be regarded as a metabolic organ and play an important role (37). It is found that the dominant bacteria in the animal gastrointestinal tract are Bacteroides and Firmicutes (38–40), which is consistent with the results of this study. Those indicate that Anemoside B4 supplementation would promote the stability of the intestinal flora of calves and have no adverse effect. In this study, the main phyla in the low diarrhea incidence group were *Firmicute* (62.55%), *Bacteroidetes* (23.17%), *Fusobacteriota* (5.97%), and *Proteobacteria* (5.87%), and the main phyla in the high diarrhea incidence group were *Firmicute* (78.93%), *Fusobacteriota* (7.75%), *Actinobacteriota* (5.99%), and *Proteobacteria* (5.15%) at 28 days of age. In which, there was no significant difference except for *Bacteroidetes*. *Firmicutes* are mainly involved in the hydrolysis of carbohydrates and proteins, and *Bacteroidetes* mainly act on steroids, polysaccharides, and bile acids (41, 42), indicating that Anemoside B4 reduced calf diarrhea incidence mainly by regulating the relative abundance of *Bacteroidetes* in the intestinal flora of calves and promoting the degradation of non-cellulose substances.

*Prevotella* is a genus of dominant bacteria that widely exists in the rumen and gastrointestinal tract of herbivores and omnivores. It plays an important role in protein decomposition. At the same time, it can also use the succinic acid pathway to ferment starch, monosaccharides, and other non-cellulosic polysaccharides to produce propylene acid, and further, maintain the glucose homeostasis in animals through the gluconeogenesis pathway (43). In this study, 45 mg/day Anemoside B4 significantly increased the relative abundance of *Prevotella* in the fecal microorganisms of calves, indicating that the addition of Anemoside B4 helps calves to digest and absorb nutrients and reduce the feed conversion rate. In addition, the relative abundance of *Sutterella* in the fecal microbial of calves fed 45 mg/day Anemoside B4 was the highest while the incidence of diarrhea was the lowest. The study found that *Sutterella* and *Bacteroides* may be closely related to the occurrence and development of antibiotic-associated diarrhea (AAD) in rats (44). The relative abundance of *Sutterella* is negatively correlated with the administration of Shen Ling Bai Zhu San (SLBZS), indicating that *Sutterella* is the main bacterium causing diarrhea after antibiotic treatment. Our result is contrary to the above results, which may be caused by differences in animal species. Another explanation may be different causes of calf diarrhea. A study showed that in the context of other inflammatory bowel diseases (IBDs), the detrimental effects of *Sutterella* seem to be specific to ulcerative colitis (UC), as the low abundance of *Sutterella* in patients with Crohn's disease (CD) (45). At present, there are few studies on the relationship between *Sutterella* and calf diarrhea, and specific reasons need to be further studied. The *Faecalibacterium prausnitzii* is the only bacterial species in the genus *Faecalibacterium*, and it is also one of the most important symbiotic bacteria in human intestinal microbes (46). Studies have shown that *F. prausnitzii* can metabolize unabsorbed carbohydrates in the intestine to produce butyrate and exert the body's anti-inflammatory effect by upregulating the expression of the Dact3 gene (47). In this study, Anemoside B4 increased the relative abundance of *Faecalibacterium* in the fecal flora of calves, of which 45 mg/day achieved the best effect. The above results indicate that Anemoside B4 can regulate the intestinal flora of calves and enhance the immunity of calves by increasing the relative abundance of *Faecalibacterium* in the intestines of calves and reducing the incidence of calf diarrhea. Thus, the improvement of calf diarrhea may be due to the intestinal microbial changes induced by Anemoside B4, including the increase of the relative abundance of *Prevotella, Sutterella*, and *Faecalibacterium*.

Gene function prediction results show that Anemoside B4 has an important influence on the metabolism of calves. Among them, the addition of Anemoside B4 in the calf can promote the processes of energy metabolism, glycan biosynthesis and metabolism, metabolism of cofactors and vitamins, and inhibit lipid metabolism, xenobiotics biodegradation, and metabolism. It has been found that intestinal microbes are closely related to the metabolism of the body, and intestinal flora can influence metabolic activity by improving the energy output of food, regulating the diet, or changing the host's metabolic pathway (48). Energy metabolism is closely related to the growth and development of the body. When the energy level cannot meet the needs of the body, it will decrease feed conversion rate and productivity. A certain promotion effect reflects the enhancement of anabolism in the anabolism, which is beneficial to the growth of calves, and there is a positive correlation between energy metabolism and the relative abundance of *Prevotella, Bacteroides*, and *Sutterella* in the intestine. *Prevotella* can degrade starch, monosaccharide, and other non-cellulose polysaccharides to produce volatile fatty acid (VFA) to provide energy for the body and promote rumen development (49), and *Bacteroides* is a bacterium that produces short-chain fatty acids. In this study, 45 mg/day Anemoside B4 increased the relative abundance of *Bacteroides*, which can promote fiber degradation to generate energy, inhibit opportunistic pathogens, protect the host from inflammation and colonic diseases, and reduce the diarrhea incidence (50, 51). Besides*, Prevotella* was positively correlated with the metabolism of cofactors and vitamins and glycan biosynthesis and metabolism. The study also found that with the increase of piglet age, the function of the intestinal flora gradually matures, glycan biosynthesis and metabolism, vitamin B biosynthesis significantly increased (52). In this study, glycan biosynthesis and metabolism, metabolism of cofactors and vitamins gradually increased with the addition of Anemoside B4, indicating that Anemoside B4 contributes to the maturation and stability of calf intestinal flora, thus significantly reducing the diarrhea incidence of calves. Soy isoflavone can affect drugs and exogenous metabolism by regulating the expression and activity of phase I cytochrome P450 (CYPs) enzymes and phase I detoxification enzymes, and have anticancer, antiobesity, and antioxidant activities (53). In this study, lipid metabolism and xenobiotics biodegradation and metabolism gradually decreased with the addition of Anemoside B4, indicating that Anemoside B4 contributes to fat deposition in calves. Lipid metabolism and xenobiotics biodegradation and metabolism were positively correlated with *Olsenella*, and negatively correlated with *Prevotella*. Thus, this is mainly due to B4 decreasing *Olsenella* and increasing the relative abundance of *Prevotella*, and the specific reasons need to be further studied.

## Conclusion

This study demonstrated that adding Anemoside B4 (45 mg/day) to the milk replacer significantly reduced the diarrhea incidence of suckling calves before 42 days of age. It also highlights that Anemoside B4 could promote the integrity and maturity of the intestinal mucosa. Based on 16S rRNA gene sequencing results, this study indicated that the addition of Anemoside B4 increased the relative abundance of *Bacteroidota* and decrease the relative abundance of *Firmicutes* in the fecal microbial of calves at 28 days of age. It was also discovered that 45 mg/day Anemoside B4 significantly increased the relative abundance of *Prevotella* at 28 days of age and *Sutterella* at 56 days of age. According to the function prediction of PICRUSt 2, Anemoside B4 could promote the processes of metabolism. The above results indicated that Anemoside B4 could protect the integrity of intestinal mucosa, change the structure of intestinal microbiota of calves, indicating the potential value of Anemoside B4 on regulating intestinal microbiota and treating intestinal diseases, alleviate diarrhea effectively.

## Data Availability Statement

The datasets presented in this study can be found in online repositories. The names of the repository/repositories and accession number(s) can be found in the article/Supplementary Material.

## Ethics Statement

The animal study was reviewed and approved by Animal Ethics Committee of IFRCAAS (AEC-IFR-CAAS, Beijing, China). Written informed consent was obtained from the owners for the participation of their animals in this study.

## Author Contributions

ML carried out this study and then did the sampling and laboratory works, and YT critically reviewed the manuscript. FH participated in the whole experiment. YB and TM did the sampling. YT, QD, and LJ designed and approved the study plan. All authors read and approved the final manuscript.

## Funding

This work was supported by Earmarked Fund for Beijing Dairy Industry Innovation Consortium of Agriculture Research System (BAIC06), and the Agricultural Science and Technology Innovation Program (CAAS-ASTIP-2017-FRI-04).

## Conflict of Interest

The authors declare that the research was conducted in the absence of any commercial or financial relationships that could be construed as a potential conflict ofinterest.

## Publisher's Note

All claims expressed in this article are solely those of the authors and do not necessarily represent those of their affiliated organizations, or those of the publisher, the editors and the reviewers. Any product that may be evaluated in this article, or claim that may be made by its manufacturer, is not guaranteed or endorsed by the publisher.
