# Supplementary material for: Effects of Anemoside B4 on Diarrhea Incidence, Serum Indices, and Fecal Microbial of Suckling Calves
Source: Front Vet Sci. 2022 Apr 28;9:851865. doi: 10.3389/fvets.2022.851865 (PMC9096840; doi:10.3389/fvets.2022.851865)
Supplement: Supplementary file 1 [file Table_1.docx]

**Supplement Table**

Supplement Table S1. Nutrient levels of milk replacers (%, dry matter basis)

| Nutrient levels^1^ | Contents |
| --- | --- |
| DM | 95.61 |
| CP | 25.21 |
| EE | 16.66 |
| Ash | 5.51 |
| NDF | 4.02 |
| ADF | 2.11 |
| Ca | 0.47 |
| P | 0.27 |

^1^Nutrient levels were measured values. DM = dry matter; CP = crude protein; EE = crude fat; NDF = neutral detergent fiber; ADF = acid detergent fiber; Ca = calcium; P = phosphorus.

Supplement Table S2. Ingredient and composition of the stater (%, dry matter basis)

| Items | Content |
| --- | --- |
| Ingredients |  |
| Corn | 30 |
| Soybean meal | 25 |
| Wheat bran | 9.5 |
| Flour | 6 |
| Soybean hulls | 6.5 |
| Cottonseed meal | 5.5 |
| Wheat shorts | 6 |
| DDGS | 5 |
| Corn germ meal | 2 |
| Limestone | 2.2 |
| CaHP0_4_ | 0.5 |
| NaCl | 0.8 |
| Premix^1^ | 1 |
| Total | 100 |
| Nutrient levels^2^ |  |
| DM | 90.25 |
| CP | 23.36 |
| EE | 4.37 |
| Ash | 11.47 |
| NDF | 34.49 |
| ADF | 11.15 |
| Ca | 1.33 |
| P | 0.60 |

^1^The premix provided the following per kg of the starter: VA 10000 IU,VD 2500 IU,VE 50 IU, Fe 90 mg, Cu 12.5 mg, Mn 30 mg, Zn 100 mg, Se 0.3 mg, I 1.0 mg, Co 0.5 mg.

^2^Nutrient levels were measured values. DM = dry matter; CP = crude protein; EE = crude fat; NDF = neutral detergent fiber; ADF = acid detergent fiber; Ca = calcium; P = phosphorus.
